# Comparative genetic characterization of CMY-2-type beta-lactamase producing pathogenic *Escherichia coli* isolated from humans and pigs suffering from diarrhea in Korea

**DOI:** 10.1186/s12941-023-00559-1

**Published:** 2023-01-19

**Authors:** Kwang-Won Seo, Kyung-Hyo Do, Min-Kyoung Shin, Woo-Kon Lee, Wan-Kyu Lee

**Affiliations:** 1 College of Veterinary Medicine, Chungbuk National University, Cheongju, 28644 Republic of Korea; 2 Laboratory of Veterinary Bacteriology and Infectious Diseases, College of Veterinary Medicine, Chungbuk National University, Cheongju, 28644 Republic of Korea; 3 Department of Microbiology, College of Medicine, Gyeongsang National University, Jinju, 52727 Republic of Korea

**Keywords:** *Escherichia coli*, Pig, Humans, Antimicrobial resistance, Third-generation cephalosporin, Plasmid-mediated AmpC

## Abstract

**Background:**

Pathogenic *Escherichia coli* are an important cause of bacterial infections in both humans and pigs and many of antimicrobials are used for the treatment of *E. coli* infection. The objective of this study was to investigate the characteristics and relationship between humans and pigs regarding third-generation cephalosporin resistance and CMY-2-producing *E. coli* in Korea.

**Results:**

All 103 third-generation cephalosporin-resistant *E. coli* isolates showed multidrug resistance. Also, except for β-lactam/β-lactamase inhibitor combinations, all antimicrobials resistant rates were higher in pigs than in humans. A total of 36 isolates (humans: five isolates; pigs: 31 isolates) were positive for the CMY-2-encoding genes and thirty-two (88.9%) isolates detected class 1 integrons with 10 different gene cassette arrangements, and only 1 isolate detected a class 2 integron. The most common virulence genes in pigs were LT (71.0%), F18 (51.6%), and STb (51.6%), while *stx2* (80.0%) was the most frequently detected gene in humans. *Stx2* gene was also detected in pigs (6.5%). Interestingly, 36 CMY-2-producing *E. coli* isolates showed a high diversity of sequence types (ST), and ST88 was present in *E. coli* from both pigs (11 isolates) and humans (one isolate).

**Conclusion:**

Our findings suggest that a critical need for comprehensive surveillance of third-generation cephalosporin resistance is necessary to preserve the usefulness of third-generation cephalosporins in both humans and pigs.

**Supplementary Information:**

The online version contains supplementary material available at 10.1186/s12941-023-00559-1.

## Introduction

*Escherichia coli* is member of a large bacterial family, *Enterobacteriaceae*, which consists of facultative anaerobic Gram-negative rods that live in the intestinal microflora of humans and animals. Although many *E. coli* are harmless commensals, pathogenic *E. coli* are an important cause of bacterial infections like colibacillosis. In humans, these strains are the foremost cause of diarrhea and hemorrhagic colitis as well as hemolytic uremic syndrome [1]. In pigs, these strains cause diarrhea with edema disease and economic loss on pig farming due to decreasing weight gain, and costs for feed supplements, vaccinations and treatments [2]. Trimethoprim-sulfamethoxazole, fluoroquinolones, and third-generation cephalosporins are the important antimicrobials for treating infections caused by pathogenic *E. coli*. Third-generation cephalosporins are widely used for the treatment of bacterial infections in both human and veterinary medicines [3]. Misuse of third-generation cephalosporins increases the emergence of extended-spectrum β-lactamase (ESBL) and AmpC β-lactamases producing *E. coli* isolates in humans and animals [4–6]. These isolates are resistant to β-lactam antibiotics because the ESBL and AmpC β-lactamases allow bacteria to hydrolyze β-lactam antibiotics. AmpC beta-lactamase genes can be located and encoded on chromosomes or plasmids [7].

The genes that encode on plasmid called plasmid-mediated AmpC beta-lactamases (pAmpC) and are often overexpressed [5, 6, 8]. Eight families of pAmpC have been described based on differences in the amino acid sequence: ACC (Ambler class C), ACT (AmpC type), CMY (cephamycin), DHA (Dhahran hospital in Saudi Arabia), FOX (cefoxitin), LAT (latamoxef), MIR (Miriam hospital in Providence), and MOX (moxalactam) [9, 10]. Of these groups, CMY-2 is the most widely and prevalent existed in *Enterobacteriaceae* including *E. coli* [7].

In pathogenic *E. coli*, antimicrobial resistant genes and virulence genes are frequently detected in isolates from both humans and pigs. Pigs are considered the primary reservoirs of pathogenic *E. coli* which can lead the contamination of food products such as pork, and human infection [11–13]. Although some studies about third-generation cephalosporins resistance of pathogenic *E. coli* have been reported in either human or pigs [4, 5, 8], a relative paucity of information exists showing a relationship between humans and pigs. Therefore, this study aimed to investigate the characteristics and relationship between pathogenic isolates from humans and pigs with third-generation cephalosporin resistance of CMY-2-producing *E. coli*.

## Materials and methods

### *Escherichia coli* isolates

Between 2008 and 2020, 392 pathogenic *E. coli* isolates were collected from 401 pigs with colibacillosis clinical case in 120 different farms, respectively. The farms consisted of different pig herds (50 to 100 sows per each herd). Samples were not collected repeatedly from the same farm. The aseptically collected intestinal contents and feces were inoculated on MacConkey agar (BD Biosciences, Sparks, MD) and Eosin methylene blue agar (BD Biosciences) and incubated at 37℃ for 20 to 24 h. Only one typical colony was selected from each sample and transferred to blood agar. Suspected colonies were identified as *E. coli* using the VITEK II system (bioMéreiux, Craponne, France). Hemolysis was also determined in blood agar (Asan Pharmaceutical, Seoul, Korea). Also, 197 strains from 197 diarrheic patients from 1981–2019 were provided by the National Culture Collection for Pathogens (NCCP; Korea, 51 strains), Gyeongsang National University Hospital Branch of the NCCP (GNUH-NCCP; Korea, 138 strains), and Kyungpook National University Hospital Branch of the NCCP (KNUH-NCCP; Korea, eight strains).

### Third-generation cephalosporin resistant *E. coli* identification

Two-hundred microliters of standardized inoculum (0.5 McFarland) of each *E. coli* isolate (collected on MacConkey agar without antimicrobial) was plated on Mueller–Hinton agar plates supplemented with 2 μg /mL cefotaxime (Sigma-Aldrich, St.Louis, MO) and incubated at 37 ℃ for 24 h to select third-generation cephalosporin-resistant *E. coli* [14]. Ultimately, a total of 103 third-generation cephalosporin-resistant *E. coli* strains (8 strains isolated from KNUH-NCCP, 15 strains isolated from GNUH-NCCP, 8 strains isolated from NCCP, and 72 strains isolated from 19 different pig farms in this study) were tested in this study (Additional file 1).

### Antimicrobial susceptibility test

All third-generation cephalosporin-resistant *E. coli* isolates were investigated for their antimicrobial resistance using the disc diffusion test with the following 19 discs (BD Biosciences): amikacin (30 μg), amoxicillin/clavulanate (20/10 μg), ampicillin (10 μg), cefazolin (30 μg), cefepime (30 μg), cefoxitin (30 μg), cephalothin (30 μg), chloramphenicol (30 μg), ciprofloxacin (5 μg), colistin (10 μg), doxycycline (30 μg), gentamicin (10 μg), kanamycin (30 μg), nalidixic acid (30 μg), neomycin (30 μg), norfloxacin (10 μg), streptomycin (10 μg), tetracycline (30 μg), and trimethoprim/sulfamethoxazole (1.25/23.75 μg). Results were interpreted according to the Clinical and Laboratory Standards Institute guidelines [15, 16]. The minimum inhibitory concentrations (MICs) for cefazolin, cephalothin, cefoxitin, cefotaxime, cefpodoxime, ceftazidime, ceftriaxone, and cefepime were determined by standard broth microdilution methods with Mueller–Hinton broth (BD Biosciences) according to the recommendations of the CLSI [15, 16]. *Escherichia coli* ATCC 25,922 strain was used the control organisms in the antimicrobial susceptibility tests. Multi-drug resistance (MDR) was defined as acquired non-susceptibility to at least 1 agent in 3 or more antimicrobial categories [17].

### Detection of β-lactamase-encoding genes

PCR amplification was conducted with primers (Table 1) for CTX-M, TEM, SHV, OXA, and pAmpC β-lactamase genes in the 103 third-generation cephalosporin-resistant *E. coli*. PCR products were sequenced using an automatic sequencer (Cosmogenetech, Seoul, Korea). The sequences were confirmed with those in the GenBank nucleotide database using the Basic Local Alignment Search Tool (BLAST) program available through the National Center for Biotechnology Information website (http://www.ncbi.nlm.nih.gov/BLAST). Therefore, 36 CMY-2-producing *E. coli* isolates were identified from 103 third-generation cephalosporin-resistant *E. coli* isolates.

**Table 1:** Primer sequences used for this study

Target gene	Sequence (5’ → 3’)	Size (bp)	References
B-lactamases			
TEM	F: CATTTCCGTGTCGCCCTTATTC	800	[18]
	R: CGTTCATCCATAGTTGCCTGAC		
SHV	F: AGCCGCTTGAGCAAATTAAAC	713	[18]
	R: ATCCCGCAGATAAATCACCAC		
OXA	F: GGCACCAGATTCAACTTTCAAG	564	[18]
	R: GACCCCAAGTTTCCTGTAAGTG		
CTX-M group 1	F: TTAGGAARTGTGCCGCTGYA	688	[18]
	R: CGATATCGTTGGTGGTRCCAT		
CTX-M group 2	F: CGTTAACGGCACGATGAC	404	[18]
	R: CGATATCGTTGGTGGTRCCAT		
CTX-M group 9	F: TCAAGCCTGCCGATCTGGT	561	[18]
	R: TGATTCTCGCCGCTGAAG		
CTX-M group 8/25	F: AACRCRCAGACGCTCTAC	326	[18]
	R: TCGAGCCGGAASGTGTYAT		
ACC	F: CACCTCCAGCGACTTGTTAC	346	[18]
	R: GTTAGCCAGCATCACGATCC		
FOX	F: CTACAGTGCGGGTGGTTT	162	[18]
	R: CTATTTGCGGCCAGGTGA		
MOX	F: GCAACAACGACAATCCATCCT	895	[18]
	R: GGGATAGGCGTAACTCTCCCAA		
CIT	F: CGAAGAGGCAATGACCAGAC	538	[18]
	R: ACGGACAGGGTTAGGATAGY		
DHA	F: TGATGGCACAGCAGGATATTC	997	[18]
	R: GCTTTGACTCTTTCGGTATTCG		
EBC	F: CGGTAAAGCCGATGTTGCG	683	[18]
	R: AGCCTAACCCCTGATACA		
GES	F: AGTCGGCTAGACCGGAAAG	399	[18]
	R: TTTGTCCGTGCTCAGGAT		
PER	F: GCTCCGATAATGAAAGCGT	520	[18]
	R: TTCGGCTTGACTCGGCTGA		
VEB	F: CATTTCCCGATGCAAAGCGT	648	[18]
	R: CGAAGTTTCTTTGGACTCTG		
Plasmid-mediated quinolone			
*qnrA*	F: TCAGCAAGAGGATTTCTCA	627	[19]
	R: GGCAGCACTATTACTCCCA		
*qnrB*	F: CGACCTGAGCGGCACTGAAT	515	[20]
	R: TGAGCAACGATGCCTGGTAG		
*qnrC*	F: GGGTTGTACATTTATTGAATC	447	[21]
	R: TCCACTTTACGAGGTTCT		
*qnrD*	F: CGAGATCAATTTACGGGGAATA	582	[22]
	R: AACAAGCTGAAGCGCCTG		
*qnrS*	F: ACCTTCACCGCTTGCACATT	571	[20]
	R: CCAGTGCTTCGAGAATCAGT		
*qepA*	F: CGTGTTGCTGGAGTTCTTC	403	[23]
	R: CTGCAGGTACTGCGTCATG		
Aminoglycoside-modifying enzymes			
*aac(3)-II*	F: TGAAACGCTGACGGAGCCTC	369	[24]
	R: GTCGAACAGGTAGCACTGAG		
*ant(2’’)-I*	F: GGGCGCGTCATGGAGGAGTT	740	[24]
	R: TATCGCGACCTGAAAGCGGC		
Chloramphenicol			
*catA1*	F: AGTTGCTCAATGTACCTATAACC	547	[25]
	R: TTGTAATTCATTAAGCATTCTGCC		
*cmlA*	F: CCGCCACGGTGTTGTTGTTATC	698	[25]
	R: CACCTTGCCTGCCCATCATTAG		
Sulfonamide			
*sul1*	F: CTTCGATGAGAGCCGGCGGC	433	[26]
	R: GCAAGGCGGAAACCCGCGCC		
*sul2*	F: CGGCATCGTCAACATAACC	722	[27]
	R: GTGTGCGGATGAAGTCAG		
Tetracyclines			
*tetA*	F: GTAATTCTGAGCACTGTCGC	956	[28]
	R: CTGCCTGGACAACATTGCTT		
*tetB*	F: CTCAGTATTCCAAGCCTTTG	414	[28]
	R: ACTCCCCTGAGCTTGAGGGG		
*tetC*	F: CCTCTTGCGGGATATCGTCC	505	[28]
	R: GGTTGAAGGCTCTCAAGGGC		
*tetD*	F: GGATATCTCACCGCATCTGC	436	[28]
	R: CATCCATCCGGAAGTGATAGC		
*tetE*	F: AAACCACATCCTCCATACGC	278	[28]
	R: AAATAGGCCACAACCGTCAG		
*tetG*	F: GCTCGGTGGTATCTCTGCTC	468	[28]
	R: AGCAACAGAATCGGGAACAC		
Integrons and cassettes			
Class 1 integron	F: GCCTTGCTGTTCTTCTACGG	558	[29]
	R: GATGCCTGCTTGTTCTACGG		
Class 1 cassettes	F: GGCATCCAAGCAGCAAG	variable	[29]
	R: AAGCAGACTTGACCTGA		
Class 2 integron	F: CACGGATATGCGACAAAAAGGT	788	[30]
	R: GTAGCAAACGAGTGACGAAATG		
Class 2 cassettes	F: CGGGATCCCGGACGGCATGCACGATTTGT	variable	[30]
	R: GATGCCATCGCAAGTACGAG		

### Molecular analysis

For detection of antimicrobial resistance genes and virulence genes, PCR amplification was performed using DNA extracted from 36 CMY-2-producing *E. coli* isolates (Table 1). DNA was extracted using QIAamp DNA Mini kit (QIAGEN, Germany) according to the protocol for bacteria provided by the manufacturer. All CMY-2-producing *E. coli* isolates were tested for resistance genes related to aminoglycosides (*aac (3)-II*, *aac(6’)-Ib*, and *ant(2’’)-I*), chloramphenicols (*cmlA* and *catA1*), quinolone (*qnrA*, *qnrB*, *qnrC*, *qnrD*, *qnrS*, and *qepA*), sulfonamides (*sul1* and *sul2*), and tetracyclines (*tetA*, *tetB*, *tetC*, *tetD*, *tetE* and *tetG*). Virulence factor genes associated with the toxins (LT, STa, STb, Stx2e, and EAST-1), fimbriae (F4, F5, F6, F18, and F41), and non-fimbrial adhesins (AIDA-1, paa, eae) were also confirmed by PCR as previously described [31].

### Plasmid replicon typing and detection of integrons and gene cassettes

For plasmid replicon typing and detection of integrons and gene cassettes, PCR amplification was performed using DNA extracted from CMY-2-producing *E. coli* isolates. The DNA was extracted as described above. The primers used in this study targeted 18 different replicons [32] and class 1 and 2 integrons (Table 1). Gene cassettes were tested for integron-positive isolates. The PCR products of the gene cassettes were sequenced as described above (Additional file 2).

### Multi-locus sequence typing (MLST)

All processes, including genomic DNA extraction, PCR amplification, Sanger sequencing, and assembly were performed by Macrogen (Macrogen, Seoul, South Korea). Genomic DNA were extracted using a QIAamp DNA Mini kit (QIAGEN, Germany). MLST was performed using partial sequences of seven house-keeping genes (*adk*, *fumC*, *gyrB*, *icd*, *mdh*, *purA* and *recA*). PCR was performed with 20 ng of genomic DNA as the template in a 30 μl reaction mixture, using Dr. MAX DNA Polymerase (Doctor Protein INC, South Korea) as follows: activation of Taq polymerase at 95 °C for 5 min; 35 cycles at 95 °C for 30 s, 52 °C for 30 s, and 72 °C for 1 min; and a final 10 min step at 72 °C. The products obtained after amplification were purified using a multiscreen filter plate (Millipore Corp, USA). Sequencing was performed using a PRISM BigDye Terminator v3.1 Cycle Sequencing Kit. The mixture was incubated at 95 °C for 5 min followed by 5 min on ice and then analyzed in an ABI PRISM 3730XL DNA analyzer (Applied Biosystems, USA). Sequence types (ST) were assigned online (http://pubmlst.org/biqsdb?db=pubmlst_ecoli_achtman_seqdef).

### Statistical and data analysis

The statistical package SPSS 23 was used for the description of antimicrobial patterns. A comparison of antimicrobial resistance rate between third-generation cephalosporin-resistant *E. coli* isolated from human and third-generation cephalosporin-resistant *E. coli* isolated from pig was conducted with the two-sample t-test. Differences were considered significant at *P* < 0.05.

## Results

### Antimicrobial resistance

The MDR patterns of third-generation cephalosporin-resistant *E. coli* isolated from humans and pigs are shown in Table 2. All 103 third-generation cephalosporin-resistant *E. coli* isolates showed MDR against three to nine classes of antimicrobial agents. Isolates from pigs showed resistance to 9 classes (48.6%), while no isolates from humans showed resistance to 9 classes. Although, in pigs, resistance rates of penicillins (100%), aminoglycosides (98.6%), β-lactam/β-lactamase inhibitor combinations (87.5%), folate pathway inhibitors (80.6%), phenicols (91.7%), quinolone (86.1%), and tetracycline (88.9%) were higher than 80%, resistance rates of penicillins (100%), β-lactam/β-lactamase inhibitor combinations (100%), and aminoglycosides (98.6%), were higher than 80% in humans. Also, fluoroquinolones, folate pathway inhibitors, phenicols, quionolone, and tetracycline resistant rates were significantly higher in pigs than in humans (*P* < 0.05).

**Table 2:** Distribution of multi-drug resistance in 103 third-generation cephalosporin-resistant *E. coli* isolated from humans and pigs in Korea

Antimicrobial resistance categories^a^	No. of third-generation cephalosporin-resistant *E. coli* isolates shown resistance (%)		
	Human	Pig	Total
Total	31 (100)	72 (100)	103 (100)
Nine of classes	0 (0.0)	35 (48.6)	35 (34.0)
AMGs, BL/BLICs, CEPs, FPIs, FQs, PCNs, PHs, Qs, TETs	0 (0.0)	35 (48.6)	35 (34.0)
Eight of classes	9 (29.0)	17 (23.6)	26 (25.2)
AMGs, BL/BLICs, CEPs, FPIs, FQs, PCNs, Qs, TETs	9 (29.0)	2 (2.8)	11 (10.7)
AMGs, BL/BLICs, CEPs, FPIs, FQs, PCNs, PHs, Qs	0 (0.0)	6 (8.3)	6 (5.8)
AMGs, BL/BLICs, CEPs, FPIs, PCNs, PHs, Qs, TETs	0 (0.0)	4 (5.6)	4 (3.9)
AMGs, BL/BLICs, CEPs, FQs, PCNs, PHs, Qs, TETs	0 (0.0)	3 (4.2)	3 (2.9)
AMGs, CEPs, FPIs, FQs, PCNs, PHs, Qs, TETs	0 (0.0)	2 (2.8)	2 (1.9)
Seven of classes	8 (25.8)	12 (16.7)	20 (19.4)
AMGs, BL/BLICs, CEPs, FPIs, PCNs, PHs, TETs	3 (9.7)	5 (6.9)	8 (7.8)
AMGs, BL/BLICs, CEPs, PCNs, PHs, Qs, TETs	0 (0.0)	6 (8.3)	6 (5.8)
AMGs, BL/BLICs, CEPs, FQs, PCNs, Qs, TETs	4 (12.9)	0 (0.0)	4 (3.9)
AMGs, BL/BLICs, CEPs, FPIs, FQs, PCNs, Qs	1 (3.2)	0 (0.0)	1 (1.0)
AMGs, CEPs, FQs, PCNs, PHs, Qs, TETs	0 (0.0)	1 (1.4)	1 (1.0)
Six of classes	9 (29.0)	5 (7.0)	15 (13.6)
AMGs, BL/BLICs, CEPs, FQs, PCNs, Qs	2 (6.5)	0 (0.0)	2 (1.9)
AMGs, BL/BLICs, CEPs, PCNs, PHs, TETs	2 (6.5)	0 (0.0)	2 (1.9)
AMGs, BL/BLICs, CEPs, PCNs, Qs, TETs	2 (6.5)	0 (0.0)	2 (1.9)
AMGs, CEPs, FPIs, PCNs, PHs, TETs	0 (0.0)	2 (2.8)	2 (1.9)
AMGs, BL/BLICs, CEPs, FPIs, PCNs, TETs	0 (0.0)	1 (1.4)	1 (1.0)
AMGs, BL/BLICs, CEPs, PCNs, PHs, Qs	1 (3.2)	0 (0.0)	1 (1.0)
AMGs, CEPs, FPIs, PCNs, Qs, TETs	0 (0.0)	1 (1.4)	1 (1.0)
AMGs, CEPs, FQs, PCNs, Qs, TETs	0 (0.0)	1 (1.4)	1 (1.0)
BL/BLICs, CEPs, FPIs, PCNs, PHs, TETs	1 (3.2)	0 (0.0)	1 (1.0)
BL/BLICs, CEPs, PCNs, PHs, Qs, TETs	1 (3.2)	0 (0.0)	1 (1.0)
Five of classes	1 (3.2)	2 (2.8)	3 (2.9)
AMGs, BL/BLICs, CEPs, FPIs, PCNs	1 (3.2)	0 (0.0)	1 (1.0)
AMGs, CEPs, PCNs, PHs, TETs	0 (0.0)	1 (1.4)	1 (1.0)
AMGs, CEPs, PCNs, PHs, Qs	0 (0.0)	1 (1.4)	1 (1.0)
Four of classes	4 (12.9)	0 (0.0)	4 (3.9)
AMGs, BL/BLICs, CEPs, PCNs	3 (9.7)	0 (0.0)	3 (2.9)
BL/BLICs, CEPs, PCNs, Qs	1 (3.2)	0 (0.0)	1 (1.0)
Three of classes	0 (0.0)	1 (1.4)	1 (1.0)
BL/BLICs, CEPs, PCNs	0 (0.0)	1 (1.4)	1 (1.0)

^a^AMGs, aminoglycosides; BL/BLICs, β-lactam/β-lactamase inhibitor combinations; CEPs, cephems; FPIs, folate pathway inhibitors; FQs, fluoroquinolones; PCNs, penicillins; PHs, phenicols; Qs, quionolones; TETs, tetracyclines

### Characteristics of CMY-2-producing *E. coli*

The phenotypic and genotypic characteristics of the 36 CMY-2-producing *E. coli* isolates (humans: five isolates; pigs: 31 isolates) among the 103 third-generation cephalosporin-resistant *E. coli* isolates are shown in Table 3. All CMY-2-producing *E. coli* isolates had high MICs for most cephalosporins. Among the 36 CMY-2-producing *E. coli* isolates, TEM-1 and OXA-1 genes were detected in 27 (75.0%) and 4 (11.1%) isolates, respectively. Also, 5 human isolates and 22 isolates recovered from pigs harbored both CMY-2 and TEM-1 genes, respectively. Tetracycline-resistance genes were detected in all CMY-2-producing *E. coli* isolates from both humans and pigs. In pigs, *tetA* (100.0%) was the most prevalent resistance gene, but in humans, *tetB* (100.0%) was predominant. Two types of aminoglycoside-modifying enzyme genes (*aac(6’)-Ib* and *aac(3)-II*) were examined, but there was no *ant(2’’)-I* gene in CMY-2-producing *E. coli* isolate from either humans or pigs. *Sul1* and *sul2* sulfonamide-resistance genes were detected in 41.7% (humans: 100.0%; pigs: 32.3%;) and 75.0% (humans: 40.0%; pigs: 80.6%) of isolates, respectively. In pigs, both *cmlA* (58.1%) and *catA1* (3.2%) chloramphenicol-resistance genes were identified; there were no chloramphenicol-resistance genes found in strains isolated from humans. The *qnrS* quinolone-resistance gene was the only quinolone-resistance gene detected in both pigs (32.3%) and humans (40.0%).

**Table 3:** Molecular characteristics of the 36 CMY-2-producing *E. coli* isolated from human and pigs in Korea

Origin	Isolate	Pathotype	Virotype	Resistance genes	Minimum inhibitory concentrations (μg/mL)^1^								Integron and gene cassettes	Plasmid replicon type
					CZ	CF	FOX	CTX	CPD	CAZ	CRO	FEP		
Human	HCT3R-12	STEC	stx1	*TEM-1, OXA-1, sul1, aac(6’)-Ib, aac(3)-II, tetB, tetE*	> 16	> 16	> 64	16	> 32	32	32	2	I (*aadA5*-*dfrA17*)	FIC, FIA, FIB, I1, HI1, Y
	HCT3R-13	STEC	stx2	*TEM-1, OXA-1, sul1, sul2, aac(6’)-Ib, aac(3)-II, tetB, tetE, qnrS*	> 16	> 16	> 64	> 64	> 32	> 128	> 128	> 16	I (*aadA5*-*dfrA17*)	FIC, FIA, FIB, Y
	HCT3R-14	STEC	stx1:stx2	*TEM-1, sul1, tetB, tetE*	> 16	> 16	8	> 64	1	0.5	64	≤ 1	I (*aadA5*-*dfrA17*)	FIIA, FIA, I1, Y
	HCT3R-16	STEC/EAEC	stx2:aggR	*TEM-1, sul1, aac(6’)-Ib, aac(3)-II, tetB, tetE, qnrS*	> 16	> 16	> 64	4	32	4	≤ 1	≤ 1	I (-)	FIA, FIB, I1, HI1, Y
	HCT3R-22	STEC	stx1:stx2	*TEM-1, sul1, sul2, aac(6’)-Ib, tetB, tetE*	> 16	> 16	8	> 64	> 32	64	> 128	> 16	–	FIA, FIB, I1, Y
Pig	CT3R-15	ETEC	F4:paa:LT:STb:EAST1	*TEM-1, sul2, cmlA, tetA, tetE, qnrS*	> 16	> 16	64	8	> 32	8	16	≤ 1	–	FIC, A/C, FIA, FIB, I1, Y
	CT3R-16	ETEC/STEC	F18:paa:LT:STa:Stx2:Stx2e	*sul2, cmlA, tetA, tetE*	> 16	> 16	> 64	4	32	4	≤ 1	≤ 1	I (*aadA5*-*dfrA17*)	FIC, FIA, FIB, I1, HI1, Y
	CT3R-18	-	–	*sul1, sul2, tetA, tetB, tetE, qnrS*	> 16	> 16	> 64	8	> 32	8	16	≤ 1	I (*aadA1*- *aadA2*- *aadB*-*cmlA6*)	A/C, FIB, I1, Y
	CT3R-22	ETEC	F4:paa:LT:STb:EAST1	*sul1, sul2, tetA, tetE*	> 16	> 16	32	8	> 32	4	8	≤ 1	I (*aadA2*-*dfrA12*)	FIC, FIB, I1, HI1, N
	CT3R-23	ETEC	F4:paa:LT:STb:EAST1	*sul2, tetA, tetE, qnrS*	> 16	> 16	32	4	> 32	4	8	≤ 1	–	FIC, FIB, I1, Y
	CT3R-24	ETEC/STEC	F18:paa:LT:STb:Stx2:Stx2e:EAST1	*sul2, cmlA, tetA, tetE*	> 16	> 16	> 64	8	> 32	16	32	≤ 1	I (-)	FIC, A/C, FIB, I1, HI1, Y
	CT3R-26	ETEC	F4:paa:LT:STb:EAST1	*sul2, cmlA, tetA, tetE*	> 16	> 16	32	4	32	8	≤ 1	≤ 1	I (-)	A/C, FIB, I1
	CT3R-28	ETEC	STa:STb:EAST1	*TEM-1, sul2, cmlA, tetA, tetE*	16	16	64	4	> 32	4	≤ 1	≤ 1	I (-)	A/C, FIB, HI1, N, HI2, Y
	CT3R-30	ETEC	F5:paa	*TEM-1, sul2, cmlA, aac(3)-II, tetA, tetB, tetE*	> 16	> 16	> 64	8	> 32	16	16	≤ 1	I (*aadA2*-*dfrA12*)	A/C, FIB, HI1, N, L/M
	CT3R-31	ETEC/STEC	F18:LT:Stx2e	*TEM-1, sul2, cmlA, tetA, tetB, tetE*	> 16	> 16	64	4	> 32	4	2	≤ 1	I (-)	FIB, I1, Y
	CT3R-32	ETEC/STEC	F18:LT:STa:Stx2e	*TEM-1, OXA-1, sul1, sul2, cmlA, aac(6’)-Ib, tetA, tetE*	> 16	> 16	64	4	> 32	8	2	≤ 1	I (-)	FIB, I1, Y
	CT3R-33	ETEC	F4:LT:STb:EAST1	*TEM-1, sul2, catA1, aac(3)-II, tetA, tetE, qnrS*	> 16	> 16	> 64	16	> 32	32	16	≤ 1	I (*aadA2*-*aadA28*-*dfrA12*)	FIC, A/C, FIA, FIB, I1, N
	CT3R-34	ETEC	F4:LT:STb:EAST1	*TEM-1, sul2, aac(3)-II, tetA, tetE, qnrS*	> 16	> 16	> 64	8	> 32	32	16	≤ 1	I (*aadA2*-*linF*)	FIC, A/C, FIA, FIB, I1, N
	CT3R-35	ETEC/STEC	F18:LT:STa:Stx2e	*cmlA, tetA, tetE*	> 16	> 16	> 64	4	> 32	8	2	≤ 1	I (*aadA1*)	FIB, I1, HI1, L/M, Y
	CT3R-36	ETEC/STEC	F18:LT:STa:Stx2e	*TEM-1, sul1, cmlA, tetA, tetE*	> 16	> 16	32	4	> 32	32	2	2	I (*aadA2*- *aadA12*- *aadA23*)	FIB, I1, HI1, L/M, Y
	CT3R-37	ETEC/STEC	F18:LT:STa:Stx2e	*TEM-1, cmlA, tetA, tetE*	> 16	> 16	64	4	32	8	2	≤ 1	I(-)	FIC, FIB, I1, HI1, L/M, Y
	CT3R-38	ETEC	F18:AIDA	*TEM-1, sul2, cmlA, tetA*	> 16	> 16	64	16	> 32	16	16	≤ 1	I (*aadA2*-*dfrA12*) II (*aadA1*-*sat2*-*dfrA1*)	A/C, I1, Y
	CT3R-39	ETEC/STEC	F18:paa:STa:Stx2e	*TEM-1, cmlA, tetA, tetE*	> 16	> 16	64	4	32	8	≤ 1	≤ 1	I (*aadA2*-*linF*)	I1, HI1, L/M, Y
	CT3R-40	ETEC	F4:F18:LT:STa:STbEAST1	*TEM-1, sul1, cmlA, tetA, tetE*	> 16	> 16	64	4	> 32	8	16	≤ 1	I (*aadA1*-*dfrA1*)	FIC, FIB, I1, HI1, L/M, Y
	CT3R-41	ETEC/STEC	F18:LT:Stx2e	*TEM-1, OXA-1, sul1, sul2, aac(6’)-Ib, tetA, tetE, qnrS*	> 16	> 16	64	4	32	8	≤ 1	≤ 1	I (*aadA1*-*dfrA1*)	FIC, FIB, I1, N, HI2
	CT3R-42	ETEC	F4:LT:STb:EAST1	*TEM-1, sul2, aac(3)-II, tetA, tetE, qnrS*	> 16	> 16	> 64	16	> 32	32	16	≤ 1	I (-)	FIC, A/C, FIA, FIB, I1, N, Y
	CT3R-43	ETEC	F18:LT:STa:STbEAST1	*TEM-1, sul1, sul2, tetA, tetE*	> 16	> 16	> 64	8	> 32	4	8	≤ 1	I (*aadA7*-*aac(3)-Id*)	FIC, A/C, FIB, I1, Y
	CT3R-44	ETEC	F4:LT:STb:EAST1	*TEM-1, sul2, aac(3)-II, tetA, tetE, qnrS*	> 16	> 16	> 64	32	> 32	128	64	≤ 1	I (-)	FIC, A/C, FIA, FIB, I1, N, Y
	CT3R-45	ETEC	AIDA:STb:EAST1	*TEM-1, sul1, sul2, tetA, tetE*	> 16	> 16	64	8	> 32	16	32	≤ 1	I (*aadA1*)	A/C, FIB, HI1, N, Y
	CT3R-46	ETEC	F4:LT:STb:EAST-1	*TEM-1, sul2, cmlA, aac(3)-II, tetA, tetE*	> 16	> 16	64	8	> 32	32	16	≤ 1	I (*aadA2*-*dfrA12*)	FIC, A/C, FIB, Y
	CT3R-47	ETEC	F4:LT:STb:EAST-1	*sul2, aac(3)-II, tetA, tetE, qnrS*	> 16	> 16	32	8	> 32	4	8	≤ 1	I (-)	FIC, A/C, FIB, I1
	CT3R-50	-	-	*sul2, tetA*	> 16	> 16	64	8	> 32	32	16	≤ 1	-	FIC, A/C, FIB, N
	CT3R-51	ETEC/STEC	F18:LT:Stx2e:paa	*TEM-1, sul2, cmlA, tetA, qnrS*	> 16	> 16	64	8	> 32	8	16	≤ 1	I (-)	A/C, I1
	CT3R-52	ETEC/STEC	F18:LT:STa:Stx2e:paa	*TEM-1, sul1, sul2, cmlA, aac(6’)-Ib, aac(3)-II, tetA, tetB, tetE*	> 16	> 16	> 64	8	> 32	16	16	≤ 1	I (*arr-3*-*aadA16*-*dfrA27*)	FIC, FIA, FIB, I1, Y
	CT3R-53	STEC	F18:Stx2e	*TEM-1, sul1, sul2, tetA, tetE*	> 16	> 16	64	4	> 32	4	2	≤ 1	I (*aadA2*-*dfrA12*)	I1, HI1, N, Y
	CT3R-55	ETEC	F18:paa:AIDA:STb:EAST1	*TEM-1, cmlA, tetA, tetE*	> 16	> 16	64	8	> 32	8	16	≤ 1	(-)	I1, N, Y

^a^*CZ* Cefazolin, *CF* Cephalothin, *FOX* Cefoxitin, *CTX* Cefotaxime, *CPD* Cefpodoxime, *CAZ* Ceftazidime, *CRO* Ceftriaxone, *FEP* Cefepime

Among the 36 CMY-2-producing *E. coli* isolates, 88.9% (humans: 4 isolates; pigs: 28 isolates) were found to have class 1 integrons and only 1 isolate harbored class 2 integrons (Table 3). Class 1 integrons harbored ten types of gene cassette arrangements, *aadA2*-*dfrA12* (five isolates), *aadA5*- *dfrA17* (four isolates), *aadA1* (two isolates), *aadA1*-*dfrA1* (two isolates), *aadA2*-*linF* (two isolates), *aadA1*-*aadA2*-*aadB*-*cmlA6* (one isolate), *aadA2*-*aadA12*-*aadA23* (one isolate), *aadA2*-*aadA28*-*dfrA12* (one isolate), *aadA7*-*aac(3)-Id* (one isolate), and *arr-3*-*aadA16*-*dfrA27* (one isolate). Twelve isolates did not carry any of the gene cassettes. The class 2 integron-containing strain had only the *aadA1*-*dfrA1*-*sat2* gene cassette arrangements. A total of 11 plasmid replicon types were detected in all 36 CMY-2-producing *E. coli* isolates (Table 3.). The most common plasmid replicon was FIB (83.3%), followed by I1 (75.0%) and Y (75.0%).

### Virulence factors

Distributions of virotypes are shown in Table 3. The most prevalent virulence genes in pigs were LT (22 isolates, 71.0%), F18 (16 isolates, 51.6%), and STb (16 isolates, 51.6%), while stx2 (four isolates, 80.0%) followed by stx1 (three isolates, 60.0%) were most frequently detected in humans. The stx2 gene was also detected in pigs (two isolates, 6.5%). ETEC (17 isolates, 54.8%) was most prevalent pathotype in pigs, followed by ETEC/STEC (11 isolates, 35.5%). But, in humans, STEC (four isolates, 80.0%) was the most prevalent pathotype; it was also identified in pigs (one isolate, 3.2%).

### Multi-locus sequence typing

**Fig. 1:**
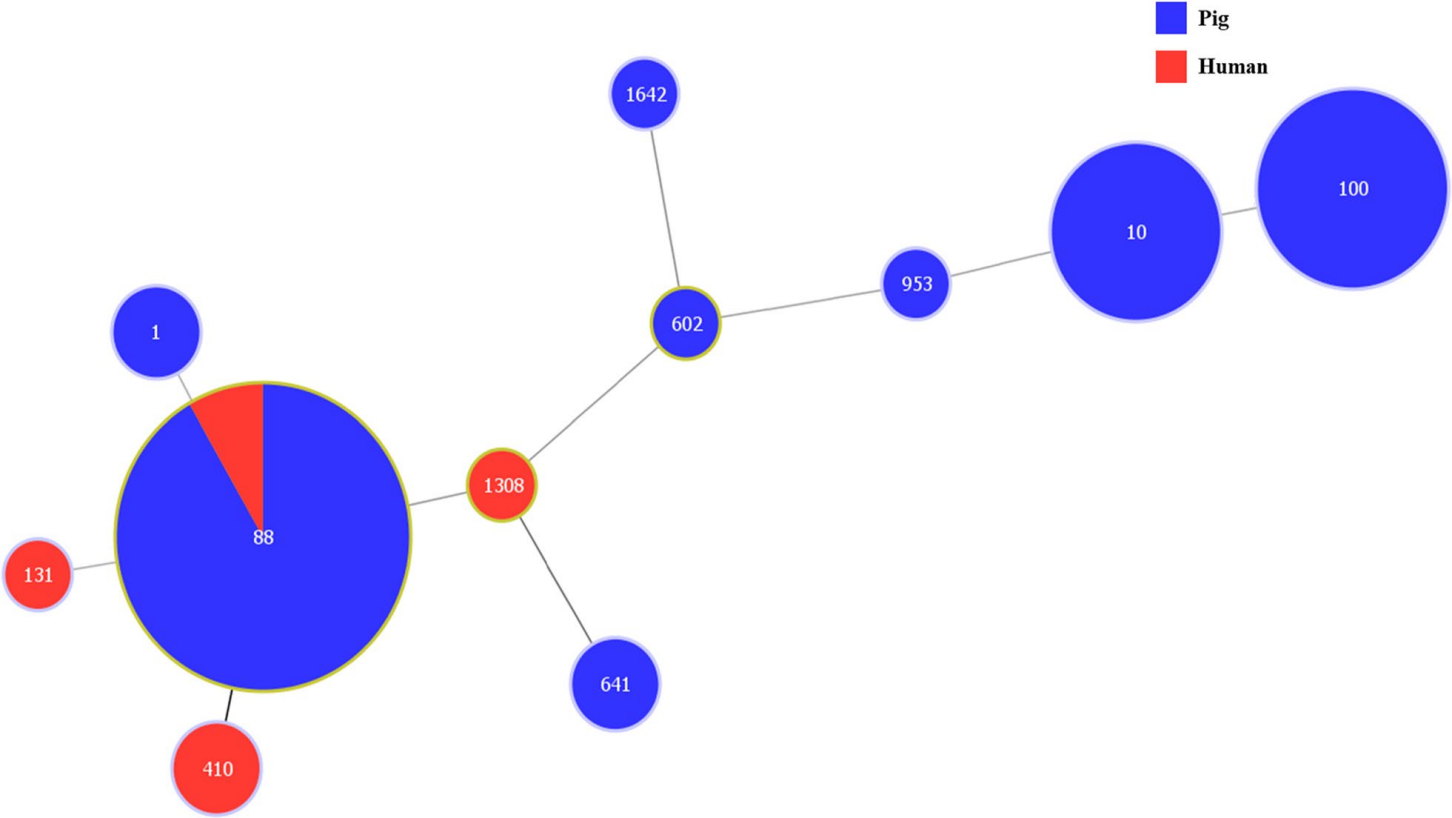
Minimum spanning tree based on sequence type of 36 CMY-2-producing *E. coli* isolated from human and pigs in Korea. Every circle represents a ST (the ST number is shown in the circle), and the size of the circle represents the number of isolates

Our collection of 36 CMY-2-producing *E. coli* isolates showed a high diversity of sequence types (ST) (Fig. 1). For isolates from human and pigs, we determined 4, and 8 different STs, respectively (Table 1). ST88 was present in *E. coli* from both pigs (11 isolates) and humans (one isolate). But several STs were only present in *E. coli* from pigs: ST100 (seven isolates), ST10 (six isolates), ST1 (two isolates), ST641 (two isolates), ST602 (one isolate), ST953 (one isolate), and ST1642 (one isolate). Also, ST410, ST131, and ST1308 were only observed in two, one, and one *E. coli* isolates, respectively, from humans. A population snapshot of 36 CMY-2-producing *E. coli* isolates, diagrammed based on a minimal spanning tree using optimized eBURST (goeBURST), based on PHYLOViZ software (www.phyloviz.net).

## Discussion

In our study, all third-generation cephalosporin-resistant *E. coli* were identified multidrug-resistant and were nonsusceptible to β-lactam antimicrobials like penicillins. These results indicate that third-generation cephalosporin-resistant *E. coli* show co-association of resistance to other classes of antimicrobials and high MDR rates. Also, although high resistance frequencies about non-beta-lactam antimicrobials were identified for aminoglycosides (98.6%), phenicols (91.7%), tetracycline (99.9%), quinolone (86.1%), and folate pathway inhibitors (80.6%) in pigs, only aminoglycoside resistance (90.3%) showed high frequency in humans. In animals, antimicrobial agents are used in large amounts to promote animal growth as well as to prevent and treat diseases [33, 34]. Therefore, the widespread use and misuse of antimicrobials in animal has resulted in the emergence of antimicrobial-resistant bacteria and it can get transferred to humans.

ESBL and pAmpC β-lactamase genes emerge when third-generation cephalosporins are overused and misused for prevention and treatment, which is a common mechanism of resistance to third-generation cephalosporins. In particular, the CMY-2-encoding gene is the most dominant pAmpC β-lactamase gene in *E. coli* isolates from both human and food producing animals. In Europe, the CMY-2-encoding gene has been detected in third-generation cephalosporin-resistant *E. coli* isolated in humans as well as in pigs [6, 35, 36]. In china, the prevalence of CMY-2-producing *E. coli* has been reported from food and domestic animals [37, 38]. Moreover, in Korea, CMY-2-encoding gene have been observed among animal and human *E. coli* isolates [5, 39, 40]. In this study, TEM-1 and OXA-1, enzymes conferring β-lactam resistance, were detected in 27 (75.0%) and 4 (11.1%) isolates, respectively. These genes are not ESBL or pAmpC β-lactamases, but can be transformed into ESBL by mutations that alter the amino acid sequence around the active site [41].

The CMY-2 gene can co-exist with other classes of antimicrobials genes in mobile genetic elements, and thus CMY-2-producing *E. coli* are commonly MDR [7, 42]. In this study, all 36 CMY-2-producing *E. coli* isolates carried a variety of antimicrobial resistance genes including *sul1*, *sul2*, *catA1*, *cmlA, aac(6’)-Ib, aac(3)-II*, *tetA*, *tetB*, *tetE*, and *qnrS*. The *tetB* gene was identified in CMY-2-producing *E. coli* isolates in both pigs and humans, which is consistent with the findings of Koga et al. and Endimiani et al. [7, 43]. The *sul1* and *sul2* genes, which encode sulfonamide-resistant dihydropteroate synthase, were identified in 15 (41.7%) and 27 (75.0%) CMY-2-producing *E. coli* isolates, respectively, and the *aac(3)-II* and *aac(6’)-Ib* genes, which encode aminoglycoside adenylyltransferase, were detected in 11 (30.6%) and 7 (19.4%) CMY-2-producing *E. coli* isolates, respectively. These genes have already been reported as major determinants of sulfonamides (*sul1* and *sul2*) and gentamicin (*aac(3)-II* and *aac(6’)-Ib*) resistance in Gram-negative bacteria [44]. Although chloramphenicol is banned in food-producing animals because of its suspected carcinogenicity [45], we found that 18 (50.0%) and one (2.8%) of 31 CMY-2-producing *E. coli* isolated from pigs carried the *cmlA* and *catA1* genes, respectively; these genes encodes a specific chloramphenicol transporter. Also, florfenicol which has been shown to have a spectrum of activity similar to that of chloramphenicol and used in veterinary medicine is related to chloramphenicol and can select for cross-resistance among bacterial pathogens [46, 47]. Therefore, although chloramphenicol is already prohibited worldwide in food animals, there is a reservoir of chloramphenicol resistance in bacteria from food animals, which can disseminate on transferable plasmids, remains a concern as chloramphenicol is a useful antibiotic for the treatment of bacterial infections in humans [48, 49]. *qnrS* genes, which encodes plasmid-mediated quinolone resistance, were identified in both pigs (32.3%) and humans (40.0%). Previous studies reported that the PMQR genes in β-lactamases-producing-*E. coli* were detected at high levels [50]. The presence of PMQR genes in β-lactamases-producing *Enterobacteriaceae* may be due to common carriage on a plasmid [51].

Integrons are genetic elements that transfer antimicrobial resistance gene, and they play an important role in conferring resistance to multiple antimicrobials [52]. In recent years, integrons have been found in β-lactamase-producing isolates of Gram-negative bacteria [53, 54]. In this study, the *aadA* gene was the most prevalent gene cassette of the integrons followed by the *dfrA* gene. These genes are also frequently detected in gene cassettes of integrons isolated from humans and pigs in Korea [55, 56]. Because β-lactamase-producing isolates harboring the *aadA* or *dfrA* or both genes showed higher antimicrobial resistance rates [57, 58], the selection of antimicrobials for the treatment of colibacillosis remains a serious concern. Also, the most common plasmid replicons were IncF plasmids including FIB (83.3%), FIC (52.8%), and FIA (33.3%). IncF plasmids are associated with important role in the worldwide emergence and spread of virulence and antimicrobial resistance determinants including extended-spectrum β-lactamases and pAmpC genes (CMY and DHA) among pathogenic *E. coli* [59].

For diagnosis and preventative measures for colibacillosis, detection of *E. coli* virulence factors is important [60]. In this study, LT, STb, F18, and F4 were detected in 22 (71.0%), 16 (51.6%), 16 (51.6%) and 11 (35.3%) CMY-2- producing *E. coli* isolates from pigs, respectively. The LT gene play a significant role in producing heat-labile enterotoxins and causing diseases [61].ST genes damage vessels and cause edema leading to high mortality in pigs [62]. Further, adhesive fimbriae gene including F4, F5, F6, F18, and F41 play important roles in allowing pathogenic *E. coli* to attach to the epithelial cells and intestinal mucosa and in causing diseases [58]. Interestingly, inactivated vaccines targeting F4 and F18 are being used in Korea [63]. The use of these vaccines could cause antigenic variations and would account for the prevalence of F4 and F18, in pigs. The *stx2* gene was also detected in isolates from both pigs (two isolates, 6.5%) and humans (four isolates, 80.0%). The *stx* gene is associated with edema disease in swine and hemolytic-uremic syndrome in humans [64–66] and the receptor for *stx2* is globotriosyl ceramide, which is found in both humans and pigs.

MLST help to identify the phylogenetic relationships among deep lineages, providing a view of the population structure of bacterial species [67]. In this study, we found eleven STs, including eight STs (ST1, ST10, ST88, ST100, ST602, ST641, ST953, and ST1642) from pigs and four STs (ST88, ST131, ST410, and ST1308) from humans. The most prevalent STs in pigs were ST88 (12 isolates), ST100 (seven isolates), and ST10 (six isolates), which are the predominant ETEC type, and are important pig pathogens in the many country (Canada, Germany, Thailand, and United States (http://mlst.warwick.ac.uk/mlst/dbs/Ecoli). In particular, ST88 was reported in both humans and pigs and it has been previously described in association with antimicrobial resistant gene like AmpC [68]. These ST is related to strains pathogenic and antimicrobial resistance and emergence of similar ST might indicates transmission between pigs and humans [69, 70].

## Conclusions

In this study, we genetically analyzed, characterized, and investigated the prevalence and relationship of third-generation cephalosporin resistance and CMY-2-producing *E. coli* isolated from humans and pigs in Korea suffering from diarrhea. To our knowledge, this is the first study to investigate the molecular characteristics and relationship between third-generation cephalosporin-resistant and CMY-2-producing *E. coli* isolated from humans and pigs in Korea. Third-generation cephalosporin resistant bacteria can get transferred to humans through the food chain and lead to treatment failure of serious infections. Therefore, a critical need for comprehensive surveillance of third-generation cephalosporin resistance is necessary to preserve the usefulness of third-generation cephalosporins in both humans and pigs.

## Supplementary Information

**Additional file 1.** Prevalence of β-lactamase-encoding genes in 103 third-generation cephalosporin-resistant *E. coli* isolates.

**Additional file 2.** Antibiotic resistance rates in 103 third-generation cephalosporin-resistant *E. coli* isolated from humans and pigs in Korea.

## Data Availability

Not applicable.
