# Supplementary material for: Comparative genetic characterization of CMY-2-type beta-lactamase producing pathogenic Escherichia coli isolated from humans and pigs suffering from diarrhea in Korea
Source: Ann Clin Microbiol Antimicrob. 2023 Jan 19;22:7. doi: 10.1186/s12941-023-00559-1 (PMC9854124; doi:10.1186/s12941-023-00559-1)
Supplement: Supplementary file 2 — Additional file 2. Antibiotic resistance rates in 103 third-generation cephalosporin-resistant E. coli isolated from humans and pigs in Korea. [file 12941_2023_559_MOESM2_ESM.docx]

Table S1. Antibiotic resistance rates in 103 third-generation cephalosporin-resistant *E. coli* isolated from humans and pigs in Korea

| Antimicrobial  resistance categories | No. of third-generation cephalosporin-resistant *E. coli* isolates shown resistance (%) | |
| --- | --- | --- |
|  | Human (n=31) | Pig (n=72) |
| Aminoglycoside | 28 (90.3) | 71 (98.6) |
| β-lactam/β-lactamase inhibitor combinations | 31 (100.0) | 63 (87.5) |
| Cephalosporins | 31 (100.0) | 72 (100.0) |
| Folate pathway inhibitors^a^ | 15 (48.4) | 58 (80.6) |
| Fluoroquinolones^a^ | 16 (51.6) | 50 (69.4) |
| Penicillins | 31 (100.0) | 72 (100.0) |
| Phenicols^a^ | 8 (25.8) | 66 (91.7) |
| Polypeptides | 0 (0.0) | 1 (1.4) |
| Quionolone^a^ | 21 (67.7) | 62 (86.1) |
| Tetracycline^a^ | 22 (71.0) | 64 (88.9) |

^a^*P*<0.05
